# Stability of resilience in times of the COVID‐19 pandemic

**DOI:** 10.1002/pmh.1560

**Published:** 2022-07-29

**Authors:** Sophie Köhne, Veronika Engert, Jenny Rosendahl

**Affiliations:** ^1^ Institute of Psychosocial Medicine, Psychotherapy and Psychooncology Jena University Hospital Jena Germany; ^2^ “Social Stress and Family Health” Research Group Max Planck Institute for Human Cognitive and Brain Sciences Leipzig Germany

## Abstract

There is disagreement among researchers regarding the conceptualization of resilience as a dynamic state or stable trait. Aiming to shed light on the state‐versus‐trait debate, we explored the stability and construct validity of four of the most frequently utilized state or trait resilience scales in a longitudinal assessment. Additionally, we examined the predictive validity of these scales. Our study was conducted before and during the COVID‐19 pandemic, which served as collectively experienced adversity. Correlations among the resilience scales and among resilience scales and Big Five personality traits were strong. All except one scale showed high test–retest correlations. Experience of an additional critical life event during the pandemic led to an increase in resilience. Other than in cross‐sectional studies, associations between resilience and psychological distress were weak, because personality and baseline psychological distress were controlled for. Nevertheless, next to personality, resilience explained additional variance in distress change. Our results show relatively high stability of resilience overall. Yet, they also confirm dynamic resilience features, suggesting that resilience change occurs with significant adversity, leading to improved adaptation. To gauge the true association between resilience and mental health, baseline levels of these variables as well as personality traits should be considered.

## INTRODUCTION

We can look back on a long tradition in resilience research with manifold conceptional approaches. At the core, these are derived from two distinct psychological domains (Luthar et al., 2000): a developmental systems perspective and a personality theory approach. The developmental systems perspective focuses on resilience in children and adolescents, who thrive despite significant adversity and trauma (Masten, 2019). The concept of resilience as personality trait is typically examined in adulthood. It stems from a psychoanalytical tradition based on the construct of ego‐resilience by Block und Block (Farkas & Orosz, 2015; Windle et al., 2011). Currently, personality psychology uses the term “trait resilience” to allow a clear distinction from the developmental systems approach (Ong et al., 2009).

Due to this ongoing dualism, there is considerable disagreement among researchers regarding the conceptualization and operationalization of resilience. In fact, for more than 50 years, there has been an ongoing debate as to whether resilience should be understood as part of a process or state or as a stable trait (Kuldas & Foody, 2021; Leys et al., 2020; Maltby et al., 2015). Conceptualized as a state, resilience refers to malleable affective‐motivational and cognitive potentials (Kuldas & Foody, 2021). As a trait, it is understood as a personality characteristic that moderates the negative effects of stress and promotes adaptation (Wagnild & Young, 1993). In attempting to bridge the conceptional discourse, and based on an extensive literature review and concept analysis of resilience research, the following definition was adopted by the U.K. Resilience and Healthy Ageing Network: “Resilience is the process of negotiating, managing and adapting to significant sources of stress or trauma. Assets and resources within the individual, their life and environment facilitate this capacity for adaptation and ‘bouncing back’ in the face of adversity. Across the life course, the experience of resilience will vary” (Windle, 2010). Most commonly, resilience is described as a positive outcome in the context of risk (Luthar et al., 2000).

Various self‐report measures to assess resilience have been developed and have undergone comprehensive evaluation in terms of psychometric properties and factor structure (Ahern et al., 2006; Fisher & Law, 2021; Maltby et al., 2015; Windle et al., 2011). Much the same as the resilience definition, these self‐report measures reflect the disagreement in conceptualization and operationalization, and no “gold standard” for assessing resilience has emerged (Windle et al., 2011).

Examining the validity of the resilience construct, a wealth of studies showed associations with personality characteristics, irrespective of whether resilience was measured as a state or trait. In detail, resilience was linked to higher extraversion, agreeableness, conscientiousness, and openness, as well as lower neuroticism (McKay et al., 2021; Oshio et al., 2018).

Again, independent of its conceptualization and the utilized measurement instrument, resilience was shown to have high predictive validity. Thus, studies on the link between resilience and mental health as an indicator of positive adaption to stress or adversities have shown strong associations in representative samples and across different patient populations (Davydov et al., 2010; Färber & Rosendahl, 2018; Färber & Rosendahl, 2020; Hu et al., 2015).

In the current study, we shed light on the state‐versus‐trait resilience debate. In a large sample of German adults, tested before and after the onset of the COVID‐19 pandemic (*N* = 488), we analyzed associations and examined the construct validity of different self‐report resilience measures. Moreover, we tested the stability and examined the predictive validity of these repeatedly assessed resilience measures for psychological distress during the first months of the pandemic. This time phase was characterized by the COVID‐19 outbreak, and the first lockdown and subsequent contact restrictions put in place to prevent the spread of COVID‐19. Thus, adversity in our sample was a collective experience, involving typical aspects of psychosocial stress such as novelty, unpredictability, and loss of control (Dickerson & Kemeny, 2004; Varga et al., 2021). The following research questions were addressed in our study:

1. Is resilience stability over time higher when measured with trait (RS25, CD‐RISC) rather than state questionnaires (BRS, BRCS)?
2. Is resilience less stable if individuals experience additional stressors during the pandemic, that is, do individuals reporting additional critical life events between T0 (pre‐pandemic) and T1 (first lockdown period in Germany) show a larger resilience increase than individuals without such critical life events?
3. Are associations of resilience and personality factors stronger if resilience is measured with trait (RS25, CD‐RISC) rather than state questionnaires (BRS, BRCS)?
4. Is resilience negatively associated with psychological distress during COVID‐19?
5. We further explored (a) whether, above and beyond personality, resilience explained significant variance in psychological distress, and (b) whether, answering to the state versus trait debate, state and trait resilience questionnaires differed in the quality of their prediction of psychological distress.

## METHODS

### Study design

This report includes two measurement time‐points of a prospective, longitudinal, observational study with altogether three measurement time‐points assessed before and during the first wave of the COVID‐19 pandemic from December 2019 to August 2020.

### Setting and participants

Participants completed an internet‐based survey assessing resilience at two measurement time‐points (T0, T1). Data collection took place prior to the first COVID‐19 related fatality (T0: December 14, 2019 to March 10, 2020), the first lockdown period (T1: April 11, 2020 to May 8, 2020), and the subsequent period of contact restrictions in Germany (T2: July 10, 2020 to August 27, 2020). The current paper focuses on the prediction of psychological distress at T1 from resilience at T0 and resilience change (T0‐T1), considering personality factors in the full participant sample. A previous paper focuses on the prediction of subjective‐emotional and physiological stress at T2 from resilience at T0 and resilience change (T0‐T1), also considering personality factors in a subsample of *N* = 80 participants (Engert et al., 2021).

Recruitment was realized via advertisement on online sites and via snowball principle. Five 20‐Euro gift cards were raffled among those who completed both surveys.

### Measures

The survey (at T0 and T1) was initially piloted on a small sample of participants before regular recruitment started. Participants had the opportunity to post comments in input fields linked to either the instructions or items.

#### Resilience

We employed four of the most frequently administered and psychometrically sound resilience questionnaires (Table 1) (Ahern et al., 2006; Hu et al., 2015; Windle et al., 2011). Although some of them are considered measures of trait resilience, each was administered at T0 *and* T1, allowing to test for the stability of the construct. Presentation order of the questionnaires was randomized per participant.

**TABLE 1:** Selected self‐report measures for the assessment of resilience

Measure	Abbreviation	Authors	Number of items	Definition of resilience (dimensions/factors)
Brief Resilient Coping Scale	BRCS	Sinclair & Wallston (2004)	4	Tendencies to cope with stressful situations and circumstances in a highly adaptive manner
Brief Resilience Scale	BRS	Smith et al. (2008)	6	Ability to bounce back or recover from stress
Connor–Davidson Resilience Scale	CD‐RISC	Connor & Davidson (2003)	25	Personal qualities that enable one to thrive in the face of adversity; five dimensions: “personal competence,” “trust/tolerance/strengthening effects of stress,” “acceptance of change and secure relationships,” “control,” “spiritual influences”
Resilience Scale	RS‐25	Wagnild & Young (1993)	25	Positive personality characteristic that moderates the negative effects of stress and promotes adaptation; two factors: “personal competence” and “acceptance of self and life”

The Resilience Scale (RS‐25) (Schumacher et al., 2005; Wagnild & Young, 1993) was conceptualized as a trait measure and consists of 25 items capturing the dimensions “Acceptance of Self and Life” and “Personal Competence.” All items are answered on a 7‐point Likert scale ranging from “disagree” (Luthar et al., 2000) to “agree” (Kuldas & Foody, 2021). A sum score is calculated (range: 25–175), with higher scores indicating greater trait resilience. In our study, RS‐25 internal consistency was *α* = 0.92 at both T0 and T1, mean inter‐item correlations were *r* = 0.31 (T0) and *r* = 0.33 (T1).

The Connor–Davidson Resilience Scale (CD‐RISC) (Connor & Davidson, 2003; Sarubin et al., 2015), which is considered a measure of trait resilience, is composed of 25 items, each rated on a 5‐point Likert scale ranging from “not true at all” (0) to “true nearly all of the time” (Windle et al., 2011). The total score ranges from 0 to 100; higher scores reflect greater resilience. CD‐RISC internal consistency was *α* = 0.91 at T0 and *α* = 0.92 at T1, mean inter‐item correlations were *r* = 0.30 (T0) and *r* = 0.32 (T1).

The Brief Resilience Scale (BRS) (Chmitorz et al., 2018; Smith et al., 2008) consists of six items rated on a five‐point Likert scale ranging from “strongly disagree” (Luthar et al., 2000) to “strongly agree” (Ong et al., 2009). Higher mean score values reflect greater resilience. The authors of the BRS make no concrete statement regarding whether they understand their scale as a trait or state measure. Yet, it was created to assess the ability to bounce back or recover from stress, which would suggest a state‐like resilience understanding. In our study, BRS internal consistency was *α* = 0.85 at both T0 and T1, and mean inter‐item correlations were *r* = 0.48 (T0) and *r* = 0.49 (T1).

The Brief Resilience Coping Scale (BRCS) (Kocalevent et al., 2017; Sinclair & Wallston, 2004) captures resilience as a dynamic state. It comprises four items that are rated on a 5‐point Likert scale ranging from “does not describe me at all” (Luthar et al., 2000) to “describes me very well” (Ong et al., 2009). Aggregated item scores range between 4 and 20, with higher scores indicating greater resilience. Internal consistency of the BRCS was *α* = 0.65 at T0 and *α* = 0.66 at T1, mean inter‐item correlations were *r* = 0.33 (T0) and *r* = 0.34 (T1).

#### Personality traits

We assessed the Big Five personality traits extraversion, neuroticism, agreeableness, conscientiousness, and openness to experience using a brief version of the NEO Five Factors Inventory (NEO‐FFI‐30) (Costa & McCrae, 1992; Körner et al., 2015). Because personality traits are considered stable across short‐term periods (Gnambs, 2014), it was only employed at T0. Participants responded to 30 items (six items per trait) on a 5‐point Likert scale ranging from “strongly disagree” (Luthar et al., 2000) to “strongly agree” (Ong et al., 2009). Mean scores are calculated for each subscale, with higher scores indicating higher levels of a respective trait.

#### Psychological distress

To assess levels of psychological distress, we applied the Brief Symptom Inventory (BSI‐18) at T0 and T1 (Derogatis, 2000; Franke et al., 2017). It consists of 18 items, with six items measuring symptoms of somatization, anxiety, and depression, respectively. Items are rated on a 5‐point Likert scale ranging from “not at all” (0) to “extremely” (4). A Global Severity Index (GSI; range 0–72) is aggregated, with higher scores indicating greater psychological distress.

#### Life events

The Life Events Checklist for DSM‐5 (LEC‐5) (Krüger‐Gottschalk et al., 2017; Weathers et al., 2013) was used to screen for experience of lifetime traumatic events. It assesses exposure to 16 events known to potentially result in distress and posttraumatic stress disorder and includes one additional item assessing any other extraordinarily stressful event. Respondents indicate varying levels of exposure to each type of event on a six‐point nominal scale (happened to me, witnessed it, learned about it, part of my job, not sure, does not apply). We included the LEC‐5 in both surveys, but at T1, it was modified to only assess exposure to events taking place between T0 and T1, with one additional item asking about personal experiences with a serious course of a SARS‐Cov‐2 infection. A binary variable contrasting “experience of a critical life event between T0 and T1” and “no critical life event between T0 and T1” was created.

#### COVID‐19 related worries

Also at T1, we applied items adapted from the German COSMO panel (Gilan et al., 2020), assessing worries about the pandemic (e.g., infection of self/others, overload of health care system, and losing one's job). Respondents rated their worries on a 7‐point Likert scale ranging from “very few” (Luthar et al., 2000) to “very many” (Kuldas & Foody, 2021). A mean score was calculated with higher scores indicating more worries.

### Bias

Sampling biases, such as self‐selection bias, may have an influence on the external validity of survey results. Therefore, to test for an influence of self‐selection, our sample was compared with existing normative data from RS‐25 (Schumacher et al., 2005), CD‐RISC (Sarubin et al., 2015), BRS (Kunzler et al., 2018), BRCS (Kocalevent et al., 2017), NEO‐FFI‐30 (Körner et al., 2015), and BSI‐18 (Franke et al., 2017) surveys. Participants were alerted to unfilled items and asked for page‐wise completion to enhance data completeness. To ensure response quality, we checked participants' completion time of the questionnaire, which was in an acceptable range for all participants. We also performed checks for rigid response patterns but did not identify any. Participants who stopped the survey prematurely or failed to respond to a sufficient number of items were excluded. Potential attrition bias from T0 to T1 was analyzed by comparing respondents and non‐respondents at T1.

### Statistical methods

Only complete data sets required for each analysis were utilized, with a maximum of 8.4% excluded participants due to missing data in one analysis (see Figure S1 for missing data pattern in the study population). Standardized mean differences for comparisons of our sample with normative data were used. To allow comparing the scores of the different resilience scales, we used the Percent of Maximum Possible (POMP) method to rescale the scores of each scale to range from 0 to 100 (Cohen et al., 1999).

Associations between the resilience scales (at T0 and T1), and between resilience scales and personality measures (at T0) were analyzed using Pearson correlations. Mean‐level stability of resilience was studied via dependent *t* tests and general linear models (GLMs); rank‐order stability was examined using Pearson correlations. We further tested whether resilience change was related to the experience of critical life events or COVID‐19‐related worries (both assessed at T1) by using GLM. Finally, we examined the association of resilience at T0 and the change of resilience (T0‐T1) on psychological distress at T1 using hierarchical multiple regression models. Models were built upon personality traits as control variables. Moreover, we controlled for psychological distress at T0, thus allowing to interpret data at T1 as change from T0. All analyses were performed with IBM SPSS statistics 27. The significance level was set to 0.05 for all analyses.

## RESULTS

### Participants

**FIGURE 1:**
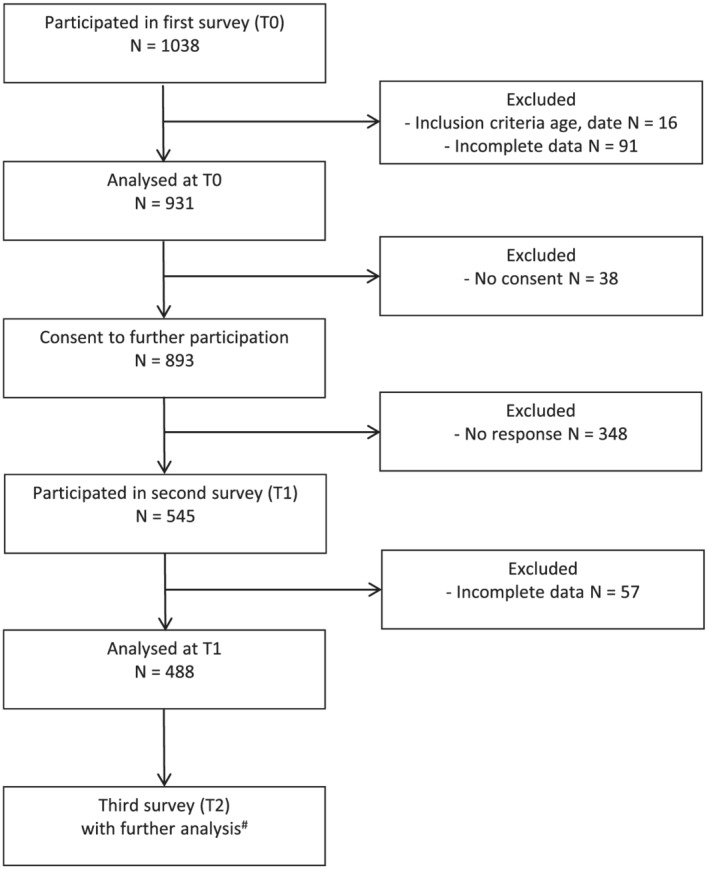
Study flow. ^#^Results were published by Engert et al. (2021)

Out of 1038 respondents of the first survey (T0), 931 were included in T0 data analysis (761 women, 81.7%; age *Mdn* = 32, *IQR* = 25–40, range = 17–78). The second survey (T1) was completed by 545 participants. Of those, 488 (403 women, 82.6%; age *Mdn* = 32, *IQR* = 25–41, range = 17–78) represented the final T1 sample (Figure 1).

Most participants were highly educated employees (Table 2). T1 respondents and non‐respondents differed significantly in education, with a larger percentage having completed college or university. Also, T1 respondents reported greater resilience (assessed at T0 with RS‐25 and BRS) and conscientiousness. Effect sizes for these group differences were very small (*d* < 0.2; Table S1).

**TABLE 2:** Descriptive data of the study sample

	Completed T0	Completed T1	Dropped out at T1	*p* ^a^	Effect size
	*N* = 931	*N* = 488	*N* = 443		
Age, *median* (IQR)	32 (25, 40)	32 (25, 41)	31 (24, 40)	0.385	0.06^b^
Sex, *N* (%)				0.661	0.03^c^
Female	761 (81.7)	403 (82.6)	358 (80.8)		
Male	167 (17.9)	83 (17.0)	84 (19.0)		
Diverse	3 (0.3)	2 (0.4)	1 (0.2)		
Education, *N* (%)				0.010	0.14^c^
Dropped out of school	5 (0.5)	3 (0.6)	2 (0.5)		
Still in school	7 (0.8)	3 (0.6)	4 (0.9)		
Completed secondary school	78 (8.4)	29 (5.9)	49 (11.0)		
Completed apprenticeship	134 (14.4)	68 (13.9)	66 (14.9)		
Completed technical school	62 (6.7)	26 (5.3)	36 (8.1)		
Completed high school	266 (28.6)	134 (27.5)	132 (29.8)		
Completed college/university	379 (40.7)	225 (46.1)	154 (34.8)		
Employment, *N* (%)				0.575	0.08^c^
High school student	8 (0.9)	2 (0.4)	6 (1.4)		
Apprenticeship	21 (2.3)	11 (2.3)	10 (2.3)		
College/university student	230 (24.7)	127 (26.0)	103 (23.3)		
Employee	561 (60.3)	291 (59.6)	270 (60.9)		
Self‐employed	42 (4.5)	20 (4.1)	22 (5.0)		
Unemployed	35 (3.8)	16 (3.3)	19 (4.3)		
Retired	20 (2.1)	13 (2.7)	7 (1.6)		
Parental leave	14 (1.5)	8 (1.6)	6 (1.4)		

^a^ *P* value for comparison of participants who completed T1 assessment versus drop‐outs; *p* from Mann–Whitney test for differences between medians (because of non‐normality of data) or *p* from chi‐square test for comparison of categorical data; IQR, interquartile range.

^b^ Effect size Cohen's *d*.

^c^ Effect sizes Cramer's *V*; can be interpreted in the same way as *r*, that is, 0.1 small, 0.3 medium, and 0.5 large effects.

In comparison with normative data, our sample showed lower CD‐RISC‐assessed resilience, greater openness (medium effect sizes), and lower psychological distress (small to medium effect sizes; Table S2).

### Main results

#### Correlations of resilience measures

Correlations of resilience measures (at T0 and T1) ranged between medium and large. While RS‐25 and CD‐RISC showed the highest (*r* > 0.8), BRS and BRCS showed the lowest correlations (*r* < 0.5) (Table S3).

#### Stability of resilience measures

Results indicate that changes in resilience were approximately normally distributed (Table 3). Regarding research question 1 (Is resilience stability over time higher when measured with trait [RS25, CD‐RISC] rather than state questionnaires [BRS, BRCS]?), mean‐level stability was highest for RS‐25, CD‐RISC, and BRCS, indicated by Cohen's *d* < 0.1. BRS showed a small mean‐level change with *d* = 0.21. The distribution of change scores was narrowest for RS‐25 and CD‐RISC and broadest for BRS (whereby a narrower change score distributions suggest higher stability). Test–retest correlations were large for all resilience measures, ranging from *r* = 0.60 to *r* = 0.85. RS‐25 and CD‐RISC showed the highest rank‐order stability from T0 to T1 (Table 3).

**TABLE 3:** Summary statistics of resilience measured via different scales and change over time

		Level at T0		Change between T0 and T1								
	*N*	*M*	*SD*	*M*	*SD*	1st	25th	50th	75th	99th	Cohen's *d* (95% CI)	*r* (95% CI)
						Percentile of distribution						
RS‐25	472	72.94	13.29	0.64	7.77	−18.67	−4.67	1.33	5.33	21.03	0.08 (−0.01; 0.17)	0.84 (0.81; 0.86)
CD‐RISC	471	66.79	13.73	0.57	7.60	−20.79	−4.00	0.00	5.00	20.28	0.08 (−0.02; 0.17)	0.85 (0.82; 0.87)
BRS	466	57.99	20.03	2.98	14.06	−33.33	−5.21	4.17	12.50	41.67	0.21 (0.12; 0.30)	0.75 (0.71; 0.79)
BRCS	468	68.39	15.38	0.07	13.86	−37.50	−6.25	0.00	6.25	37.50	0.01 (−0.09; 0.10)	0.60 (0.53; 0.65)

*Note*: Resilience measures were transformed using the Percent of Maximum Possible (POMP) method to range from 0 to 100. Resilience change (T0‐T1) is shown as means and effect sizes (Cohen's *d* with 95% CI); positive values indicate a resilience increase; *r* represents Pearson correlation coefficients between resilience measures at T0 and T1.

Abbreviations: BRCS, Brief Resilience Coping Scale; BRS, Brief Resilience Scale; CD‐RISC, Connor–Davidson Resilience Scale; RS‐25, Resilience Scale.

With respect to research question 2 (Is resilience less stable if individuals experience additional stressors during the pandemic, i.e., do individuals reporting additional critical life events between T0 and T1 show a larger resilience increase than individuals without such critical life events?), we found that experience of additional critical life events between T0 and T1 was associated with a larger resilience increase. This difference in resilience change was significant for CD‐RISC and BRS (Table S4). COVID‐19‐related worries at T1 were unassociated with resilience change.

#### Correlations of resilience with personality measures and psychological distress

All resilience measures were negatively associated with neuroticism and positively related to extraversion and conscientiousness (medium to large correlations). Addressing research question 3 (Are associations of resilience and personality factors stronger if resilience is measured with trait [RS25, CD‐RISC] rather than state questionnaires [BRS, BRCS]?), results showed that the strengths of associations were fluctuating, and correlations of resilience trait measures with Big Five were not consistently stronger than for state questionnaires. Moreover, small to medium negative correlations emerged for the association of resilience and psychological distress (Table 4).

**TABLE 4:** Pearson correlation coefficients of the resilience measures with personality traits

	Resilience			
	RS‐25	CD‐RISC	BRS	BRCS
Personality traits				
Neuroticism	−0.69 (870)	−0.69 (870)	−0.67 (870)	−0.46 (869)
Extraversion	0.51 (871)	0.60 (871)	0.44 (871)	0.42 (870)
Openness	0.07 (871)	0.10 (871)	0.01 (871)	0.18 (870)
Agreeableness	0.13 (870)	0.11 (870)	0.08 (870)	0.14 (869)
Conscientiousness	0.50 (871)	0.47 (871)	0.31 (871)	0.33 (870)

*Note*: *P* < 0.001 for all correlations. Number of analyzed participants in parentheses.

Abbreviations: BRCS, Brief Resilience Coping Scale; BRS, Brief Resilience Scale; CD‐RISC, Connor–Davidson Resilience Scale; RS‐25, Resilience Scale.

#### Prediction of mental health from resilience and personality measures

The exploration of research question 4 (Is resilience negatively associated with psychological distress during COVID‐19?) yielded negative bivariate associations of resilience (at T0 and T1) and psychological distress (at T1) that were medium in size for all resilience scales, except for the BRCS, which showed small associations (Table S5). We further tested the predictive validity of the resilience measures on psychological distress at T1 using more complex hierarchical regression models. As primary predictors, the model included resilience at T0 and resilience change from T0 to T1. Baseline levels of psychological distress were considered as covariate. Given the strong associations between measures of resilience and personality, the Big Five subscales were also included into the model. In answering research question 5a, this allowed gauging the influence of resilience above and beyond the influence of personality. Psychological distress at T0 explained the largest amount of variance (46%) in psychological distress at T1. Extraversion was another significant predictor, also negatively associated with resilience. Additionally, change in resilience from T0 to T1 was associated with psychological distress, such that a steeper decline in resilience resulted in more psychological distress. This held true for all resilience measures except the BRCS. In the full hierarchical model, resilience at T0 was also negatively related to psychological distress, if measured via RS‐25 or BRS (Table S6, cf. research question 5b [Do state and trait resilience questionnaires differ in the quality of their prediction of psychological distress?]).

Sensitivity analysis showed that results remained stable if a psychological distress change score residualized for T0 levels was entered as dependent variable (Table S7). If personality traits were removed from the model, resilience showed stronger associations with psychological distress, and resilience at T0 was a significant predictor in the full model for all scales except the BRCS. This additional analysis confirms the strong dependency of resilience on personality traits (Table S8). Finally, when excluding psychological distress at T0 from the model, neuroticism in addition to extraversion turned out to be positively associated with psychological distress. Resilience at T0 and resilience change from T0 to T1 remained significant predictors of psychological distress at T1 (Table S9).

## DISCUSSION

In one of the few longitudinal assessment of resilience, we explored associations and examined the construct validity of different resilience measures, designed to capture resilience either as a state or trait. Additionally, we examined the stability and predictive validity of these resilience measures. Our study was conducted before and during the COVID‐19 pandemic. Compared with other resilience studies, the COVID‐19 outbreak and lockdown allowed to investigate resilience change in response to a unique, collectively experienced adversity in all of our participants.

We found large correlations among the resilience scales, as well as among resilience scales and Big Five personality traits. Regarding stability assessments, RS‐25 and CD‐RISC showed the highest test–retest correlations. Interestingly, experiencing an additional critical life event during the pandemic led to an *increase* in resilience, which was best depicted by BRS and CD‐RISC. All resilience measures negatively predicted COVID‐19 induced psychological distress. This effect was found above and beyond the contribution of personality.

The strong positive correlations between the resilience scales indicate that, despite different conceptualizations as either state or trait measures, a common construct is assessed. Here, it is interesting to note that the highest correlation was found between scales that conceptualize resilience as a trait (RS‐25, CD‐RISC). Also, as is reliably shown in the literature (McKay et al., 2021; Oshio et al., 2018), correlations with personality traits (particularly neuroticism and extraversion) were high.

Independent of whether a scale operationalized resilience as a state or trait, mean‐level and rank‐order stability from T0 (pre‐pandemic) to T1 (after COVID‐19 outbreak) were high. In fact, test–retest correlations for resilience were well within the range typically found for short‐term stability of Big Five personality factors (*r* = 0.7–0.9) (Costa & McCrae, 1986). This means that, despite a significant stressor taking place between the two surveys, resilience acted in a trait‐like manner. However, mean‐level stability was lower if participants suffered from *additional* adversity between T0 and T1. In this case, the BRS, which was created to assess resilience as a modifiable state, showed the largest mean‐level change. RS‐25 and CD‐RISC showed less, yet significant change. The BRCS was least change sensitive.

These stability findings raise two main questions. First, why did we find change only if participants reported individual experience of adversity in addition to the diverse stressors associated with the COVID‐19 outbreak? Given the wealth of literature showing significant emotional strain (Petzold et al., 2020; Robillard et al., 2020; Veer et al., 2021) and physiological stress (Engert et al., 2021) in reaction to the pandemic, we know that it was a sufficiently salient incidence to trigger resilience change—provided resilience actually is a dynamic construct. The answer may be that resilience is relatively stable overall, which is in accord with its high association with personality traits. However, with an increasing number of stressors, a threshold may eventually be exceeded, and change may be triggered.

The second question concerns the direction of change. Given cumulative adversity, why was change in resilience positive rather than negative? In general, theory claims that significant experience of stress may boost resilience, which, in turn, allows successful adaptation to adversity, and thus reduces psychological strain (Kalisch et al., 2017). Our data are in line with this dynamic understanding of resilience: Change occurs with significant adversity, and positive change is linked to improved adaptation.

Regarding the predictive validity of the resilience scales, we showed that if personality is considered, resilience explains significant, yet little, additional variance in change in psychological distress over the first months of the pandemic. This is true for all except one of the scales (BRCS).

Cross‐sectional studies typically detect much stronger correlations (*r* = 0.4) between resilience and psychological distress (Färber & Rosendahl, 2018; Färber & Rosendahl, 2020). We suggest two main reasons for this discrepancy. First, the majority of cross‐sectional studies focused on resilience alone, without considering personality traits. Likewise, in our data, bivariate associations between resilience and psychological distress were considerably higher than in the complex models including personality. Second, in cross‐sectional studies, resilience and psychological distress are only measured once and at the same time‐point. Again, in our data, bivariate associations of resilience and distress assessed simultaneously were comparable with those reported in meta‐analyses (Färber & Rosendahl, 2018; Färber & Rosendahl, 2020). It is noteworthy that psychological distress was overall very stable; the strongest predictor of psychological distress at T1 was the pre‐pandemic baseline in psychological distress measured at T0.

The strong covariance with personality that we found has been discussed previously. In fact, it was hypothesized that once personality has been accounted for, resilience scales do not explain additional variance in emotional disturbance and adaptation (Huey & Weisz, 1997; Waaktaar & Torgersen, 2010). Yet, our data confirm that, while resilience explains a relatively minor amount of variance in psychological distress, it does make an independent contribution above and beyond the Big Five. Thus, while resilience may not be independent of personality factors, its treatment as a construct in its own right seems warranted.

There are several limitations to the current study. First, as often in survey studies, sampling bias due to participant self‐selection may have influenced the external validity of our results: More than 80% of the sample was female, and more than twice as many participants as in the German population were highly educated (i.e., completed high school or college/university) (Educational Attainment of the Population in Germany, 2020). Our sample also reported more openness and less psychological distress than the general population. Second, 48% of the T0 sample did not complete the survey at T1, potentially leading to attrition bias. However, a comparison of T1 respondents and non‐respondents showed that the two groups only differed regarding resilience and conscientiousness ratings. Third, the time period from T0 to T1 was relatively short (*Mdn* = 82 days, *IQR* 58–91 days). One might argue that meaningful intraindividual change in resilience only emerges after longer time periods. Nevertheless, research has shown that particularly the first months of the pandemic were associated with significant stress (Veer et al., 2021), likely due to the unpredictability and uncontrollability of the situation (Dickerson & Kemeny, 2004). Fourth, calculating difference scores for resilience change in predicting psychological distress at T1 may have led to reliability problems (Edwards, 2002). This issue could have been solved by use of structural equation models with specification of latent difference scores (Henk & Castro‐Schilo, 2016; McArdle & Nesselroade, 1994). However, in order to address several explorative research questions, we nevertheless opted for a regression approach (Fan et al., 2016). Fifth, a general problem of any trait concept (personality or resilience) assessed using questionnaires is proneness to mood effects (Querengässer & Schindler, 2014). Yet, specifically measures of personality traits have demonstrated relative stability despite significant changes in depression scores (Santor et al., 1997). Sixth, participants may have been fatigued by the amount of survey items, leading to decreased data quality (Galesic & Bosnjak, 2009). Because surveys were presented in randomized sequence, however, any measurement error due to survey length should be balanced across the utilized measures. Finally, our study is restricted to a variable‐centered research approach, which focuses on explaining relationships between variables of interest in a population (Howard & Hoffman, 2018). A person‐centered approach would additionally allow to identify subgroups or clusters of individuals with distinct patterns of responses. We encourage future research to follow such a person‐centered approach in studying resilience.

## CONCLUSIONS

In one of the few longitudinal assessments of the construct, we examined how well resilience predicted change in psychological distress in the face of adversity. As a model situation of adversity, all participants experienced the early phases of the COVID‐19 pandemic and subsequent lockdown measures in Germany. Other than in cross‐sectional studies, associations between resilience and psychological distress were relatively weak, because we controlled for personality and baseline psychological distress. Nevertheless, next to personality, resilience explained additional variance in distress change.

Our results allow to make useful recommendations for future studies. To gauge the true association between resilience and mental health outcomes, baseline levels of these variables should be considered. Also, to guide the choice of a resilience scale best suitable for a specific research focus, one may wish to consider that, true to their conceptualization, RS‐25 and CD‐RISC showed the highest mean‐level and rank‐order stability, and BRS was most sensitive to depict change.

## CONFLICT OF INTEREST

The authors declare that they have no conflict of interest.

## ETHICS STATEMENT

Our research was approved by the ethics committee of the Jena University Hospital, Germany (#2019‐1609‐Bef., December 12, 2019).

## Supporting information


**Table S1.** Resilience, personality traits, and psychological distress at T0 in the study sample
**Table S2.** Comparison of the study sample at T0 to normative data
**Table S3:** Pearson correlation coefficients of the resilience measures at T0 (above the diagonal) and T1 (below the diagonal)
**Table S4.** Stability of resilience (T0‐T1) for participants with/without experience of a critical life event between T0 and T1
**Table S5:** Pearson correlation coefficients of the resilience measures at T0 and T1 with psychological distress at T1
**Table S6.** Model indices of hierarchical multiple regression analyses for psychological distress (Global Severity Index of BSI‐18) at T1 with psychological distress at T0 (model 1), personality traits (model 2), resilience at T0 (model 3a) or/and changes in resilience from T0 to T1 (models 3b and 4) as explanatory covariates
**Table S7.** Model indices of hierarchical multiple regression analyses for residual change scores in psychological distress (Global Severity Index of BSI‐18) with personality traits at T0 (model 1), resilience at T0 (model 2a) or/and changes in resilience from T0 to T1 (models 2b and 3) as explanatory covariates
**Table 8.** Model indices of hierarchical multiple regression analyses for psychological distress (Global Severity Index of BSI‐18) at T1 with psychological distress at T0 (model 1), resilience at T0 (model 2a) or/and changes in resilience from T0 to T1 (models 2b and 3) as explanatory covariates
**Table S9.** Model indices of hierarchical multiple regression analyses for psychological distress (Global Severity Index of BSI‐18) at T1 with personality traits at T0 (model 1), resilience at T0 (model 2a) or/and changes in resilience from T0 to T1 (models 2b and 3) as explanatory covariates
**Figure S1.** Missing data pattern in the study population included at T0 (N = 488). T0 denotes the first measurement time point and t1 the second measurement time‐point. Numbers on the left side indicate the frequency with which this missing data pattern occurs. Numbers in the bottom indicate the number of missing data of the respective variable. Numbers on the right side indicate the number of missing variables in the respective missing data pattern. Color coding: green, not missing; red, missing. Abbreviations: BRCS, Brief Resilience Coping Scale; BRS, Brief Resilience Scale; BSI, Brief Symptom Inventory; CD‐RISC, Connor‐Davidson Resilience Scale; NEOFFI, NEO Five Factors Inventory; RS‐25, Resilience Scale.

## Data Availability

Data are accessible upon reasonable request. The materials used in these studies are widely available.
