# Supplementary material for: Stability of resilience in times of the COVID‐19 pandemic
Source: Personal Ment Health. 2022 Jul 29:10.1002/pmh.1560. Online ahead of print. doi: 10.1002/pmh.1560 (PMC9353390; doi:10.1002/pmh.1560)
Supplement: Supplementary file 1 — Table S1. Resilience, personality traits, and psychological distress at T0 in the study sample Table S2. Comparison of the study sample at T0 to normative data Table S3: Pearson correlation coefficients of the resilience measures at T0 (above the diagonal) and T1 (below the diagonal) Table S4. Stability of resilience (T0‐T1) for participants with/without experience of a critical life event between T0 and T1 Table S5: Pearson correlation coefficients of the resilience measures at T0 and T1 with psychological distress at T1 Table S6. Model indices of hierarchical multiple regression analyses for psychological distress (Global Severity Index of BSI‐18) at T1 with psychological distress at T0 (model 1), personality traits (model 2), resilience at T0 (model 3a) or/and changes in resilience from T0 to T1 (models 3b and 4) as explanatory covariates Table S7. Model indices of hierarchical multiple regression analyses for residual change scores in psychological distress (Global Severity Index of BSI‐18) with personality traits at T0 (model 1), resilience at T0 (model 2a) or/and changes in resilience from T0 to T1 (models 2b and 3) as explanatory covariates Table 8. Model indices of hierarchical multiple regression analyses for psychological distress (Global Severity Index of BSI‐18) at T1 with psychological distress at T0 (model 1), resilience at T0 (model 2a) or/and changes in resilience from T0 to T1 (models 2b and 3) as explanatory covariates Table S9. Model indices of hierarchical multiple regression analyses for psychological distress (Global Severity Index of BSI‐18) at T1 with personality traits at T0 (model 1), resilience at T0 (model 2a) or/and changes in resilience from T0 to T1 (models 2b and 3) as explanatory covariates Figure S1. Missing data pattern in the study population included at T0 (N = 488). T0 denotes the first measurement time point and t1 the second measurement time‐point. Numbers on the left side indicate the frequency with which this missing data pa [file PMH-9999-0-s001.docx]

**Supplementary Material**

[Supplementary Table 1. Resilience, personality traits, and psychological distress at T0 in the study sample 2](#_Toc106186229)

[Supplementary Table 2. Comparison of the study sample at T0 to normative data. 3](#_Toc106186230)

[Supplementary Table 3: Pearson correlation coefficients of the resilience measures at T0 4](#_Toc106186231)

[Supplementary Table 4. Stability of resilience (T0-T1) for participants with/without experience of a critical life event between T0 and T1 5](#_Toc106186232)

[Supplementary Table 5: Pearson correlation coefficients of the resilience measures at T0 and T1 with psychological distress at T1. 6](#_Toc106186233)

[Supplementary Table 6. Model indices of hierarchical multiple regression analyses for **psychological distress (Global Severity Index of BSI-18) at T1** with psychological distress at T0 (model 1), personality traits (model 2), resilience at T0 (model 3a) or/and changes in resilience from T0 to T1 (models 3b and 4) as explanatory covariates 7](#_Toc106186234)

[Supplementary Table 7. Model indices of hierarchical multiple regression analyses for **residual change scores in psychological distress (Global Severity Index of BSI-18)** with personality traits at T0 (model 1), resilience at T0 (model 2a) or/and changes in resilience from T0 to T1 (models 2b and 3) as explanatory covariates 9](#_Toc106186235)

[Supplementary Table 8. Model indices of hierarchical multiple regression analyses for **psychological distress (Global Severity Index of BSI-18) at T1** with psychological distress at T0 (model 1), resilience at T0 (model 2a) or/and changes in resilience from T0 to T1 (models 2b and 3) as explanatory covariates 10](#_Toc106186236)

[Supplementary Table 9. Model indices of hierarchical multiple regression analyses for **psychological distress (Global Severity Index of BSI-18) at T1** with personality traits at T0 (model 1), resilience at T0 (model 2a) or/and changes in resilience from T0 to T1 (models 2b and 3) as explanatory covariates 11](#_Toc106186237)

[Supplementary Figure 1. Missing data pattern in the study population included at T0 (*N* = 488). 12](#_Toc106186238)

# Supplementary Table 1. Resilience, personality traits, and psychological distress at T0 in the study sample

|  | Completed T0 | Completed T1 | Dropped out at T1 | Cohen´s *d*  (95% CI)^#^ |
| --- | --- | --- | --- | --- |
|  | *N* = 931^a^ | *N* = 488^b^ | *N* = 443^c^ |  |
| Resilience, *M* (*SD*) |  |  |  |  |
| **RS-25** | **132.74 (20.57)** | **134.49 (19.80)** | **130.80 (21.25)** | **0.18 (0.05; 0.31)** |
| CD-RISC | 66.39 (13.62) | 66.74 (13.71) | 65.99 (13.52) | 0.06 (-0.07; 0.18) |
| **BRS** | **3.25 (0.80)** | **3.32 (0.79)** | **3.17 (0.80)** | **0.19 (0.06; 0.32)** |
| BRCS | 14.79 (2.62) | 14.94 (2.46) | 14.62 (2.79) | 0.12 (-0.01; 0.25) |
| Personality traits |  |  |  |  |
| Neuroticism | 15.79 (5.37) | 15.51 (5.33) | 16.13 (5.40) | 0.12 (-0.02; 0.25) |
| Extraversion | 19.39 (3.81) | 19.42 (3.72) | 19.36 (3.92) | 0.02 (-0.12; 0.15) |
| Openness | 20.71 (4.64) | 20.93 (4.64) | 20.44 (4.63) | 0.11 (-0.03; 0.24) |
| Agreeableness | 23.09 (3.91) | 23.21 (3.93) | 22.94 (3.88) | 0.07 (-0.06; 0.20) |
| **Conscientiousness** | **24.03 (3.54)** | **24.25 (3.55)** | **23.78 (3.51)** | **0.13 (0.00; 0.27)** |
| Psychological distress |  |  |  |  |
| Anxiety | 0.78 (1.62) | 0.77 (1.59) | 0.79 (1.66) | -0.01 (-0.15; 0.12) |
| Depression | 0.73 (1.78) | 0.63 (1.69) | 0.85 (1.88) | -0.12 (-0.25; 0.01) |
| Somatization | 0.39 (1.07) | 0.36 (1.03) | 0.42 (1.11) | -0.05 (-0.19; 0.08) |
| Global Severity Index | 1.90 (3.73) | 1.76 (3.58) | 2.06 (3.90) | -0.08 (-0.21; 0.05) |

^#^ Effect size for comparison of participants who completed T1 assessment vs. drop-outs; Positive values of Cohen’s d indicate higher scores of completers, negative values represent lower scores.

# Supplementary Table 2. Comparison of the study sample at T0 to normative data.

|  | Cohen´s *d* | 95% *CI* | *p* |
| --- | --- | --- | --- |
| Resilience |  |  |  |
| RS-25 | -0.05 | -0.12; 0.03 | 0.236 |
| **CD-RISC** | **-0.56** | **-0.72; -0.41** | **< 0.001** |
| BRS | -0.12 | -0.20; -0.03 | 0.009 |
| BRCS | 0.02 | -0.06; 0.09 | 0.669 |
| Personality (NEO-FFI-30) |  |  |  |
| Neuroticism | 0.15 | 0.07; 0.23 | < 0.001 |
| Extraversion | -0.07 | -0.15; 0.01 | 0.100 |
| **Openness** | **0.59** | **0.51; 0.67** | **< 0.001** |
| Agreeableness | 0.09 | 0.01; 0.17 | 0.024 |
| Conscientiousness | 0.08 | -0.01; 0.16 | 0.053 |
| Psychological distress (BSI-18) |  |  |  |
| **Anxiety** | **-0.28** | **-0.35; -0.20** | **< 0.001** |
| **Depression** | **-0.35** | **-0.43; -0.27** | **< 0.001** |
| **Somatization** | **-0.47** | **-0.55; -0.39** | **< 0.001** |
| **Global Severity Index** | **-0.41** | **-0.49; -0.34** | **< 0.001** |

BRCS, Brief Resilience Coping Scale; BRS, Brief Resilience Scale; BSI-18, Brief Symptom Inventory; CD-RISC, Connor-Davidson Resilience Scale; CI, Confidence interval; Cohen´s d, Standardized mean difference between study sample and norm data; NEO-FFI-30, NEO Five Factors Inventory; RS-25, Resilience Scale. Positive values of Cohen’s *d* indicate higher scores of the study sample in comparison to the normative sample, negative values represent lower scores.

Supplementary Table 3: Pearson correlation coefficients of the resilience measures at T0 (above the diagonal) and T1 (below the diagonal)

|  | RS-25 | CD-RISC | BRS | BRCS |  |  |  |
| --- | --- | --- | --- | --- | --- | --- | --- |
| RS-25 |  | 0.82 (911) | 0.60 (911) | 0.61 (909) |  |  | ≥ 0.7 |
| CD-RISC | 0.85 (466) |  | 0.64 (907) | 0.68 (900) |  |  | ≥ 0.5 |
| BRS | 0.65 (469) | 0.63 (466) |  | 0.41 (903) |  |  | ≥ 0.3 |
| BRCS | 0.64 (468) | 0.70 (466) | 0.49 (467) |  |  |  | < 0.3 |

*P* <.001 for all correlations. Number of analyzed participants in parentheses. BRCS, Brief Resilience Coping Scale; BRS, Brief Resilience Scale; CD-RISC, Connor-Davidson Resilience Scale; RS-25, Resilience Scale.

# Supplementary Table 4. Stability of resilience (T0-T1) for participants with/without experience of a critical life event between T0 and T1

|  | No life event from T0 to T1 | | |  | Life event from T0 to T1 | | |  |  |  |
| --- | --- | --- | --- | --- | --- | --- | --- | --- | --- | --- |
|  |  | Level at T0 | Change between T0 and T1 |  |  | Level at T0 | Change between T0 and T1 |  |  |  |
|  | *N* | *M* (95% *CI*) | *M* (95% *CI*) | *r* (95% *CI*) | *N* | *M* (95% *CI*) | *M* (95% *CI*) | *r* (95% *CI*) | *p*  (M_diff_)^a^ | *p*  (*r*_diff_)^b^ |
| RS-25 | 243 | 73.08 (71.40; 74.76) | 0.11 (-0.87; 1.08) | 0.83 (0.79; 0.86) | 229 | 72.78 (71.05; 74.51) | 1.21 (0.20; 2.23) | 0.84 (0.80; 0.88) | 0.122 | 0.309 |
| CD-RISC | 243 | 66.54 (64.81; 68.27) | -0.14 (-1.14; 0.85) | 0.82 (0.78; 0.86) | 228 | 67.06 (65.27; 68.85) | 1.33 (0.39; 2.27) | 0.87 (0.84; 0.90) | 0.036 | 0.032 |
| BRS | 241 | 60.49 (57.98; 63.01) | 1.38 (-0.41; 3.18) | 0.73 (0.66; 0.78) | 225 | 55.31 (52.71;57.92) | 4.69 (2.87; 6.50) | 0.77 (0.71; 0.82) | 0.011 | 0.131 |
| BRCS | 242 | 67.43 (65.49; 69.37) | -0.46 (-2.21; 1.28) | 0.58 (0.49; 0.66) | 226 | 69.41 (67.41; 71.42) | 0.64 (-1.19; 2.46) | 0.61 (0.52; 0.68) | 0.391 | 0.324 |

BRCS, Brief Resilience Coping Scale; BRS, Brief Resilience Scale; CD-RISC, Connor-Davidson Resilience Scale; CI, confidence interval; RS-25, Resilience Scale. Positive values indicate an increase of resilience from T0 to T1; changes in resilience from T0 to T1 are shown as means with 95% CI. Positive values indicate an increase of resilience from T0 to T1. ^a^ *p* for difference of change between subgroups (no life event vs. life event) from GLM; ^b^ *p* for difference of correlation coefficients.

# Supplementary Table 5: Pearson correlation coefficients of the resilience measures at T0 and T1 with psychological distress at T1.

|  | Resilience at T0 - Psychological distress at T1 | Resilience at T1 - Psychological distress at T1 |  |  |  |
| --- | --- | --- | --- | --- | --- |
| RS-25 | -0.44 (462) | -0.50 (463) |  |  | ≤ -0.1 |
| CD-RISC | -0.41 (458) | -0.45 (463) |  |  | ≤ -0.3 |
| BRS | -0.41 (457) | -0.46 (463) |  |  | ≤ -0.5 |
| BRCS | -0.21 (457) | -0.28 (462) |  |  |  |

*P* <.001 for all correlations. Number of analyzed participants in parentheses. BRCS, Brief Resilience Coping Scale; BRS, Brief Resilience Scale; CD-RISC, Connor-Davidson Resilience Scale; RS-25, Resilience Scale.

# Supplementary Table 6. Model indices of hierarchical multiple regression analyses for **psychological distress (Global Severity Index of BSI-18) at T1** with psychological distress at T0 (model 1), personality traits (model 2), resilience at T0 (model 3a) or/and changes in resilience from T0 to T1 (models 3b and 4) as explanatory covariates

|  | RS-25 (*N* = 448) | | | | | | | | | |
| --- | --- | --- | --- | --- | --- | --- | --- | --- | --- | --- |
|  | Step 1 | | Step 2 | | Step 3a | | Step 3b | | Step 4 | |
| Predictors | β | *p* | β | *p* | β | *p* | β | *p* | β | *p* |
| GSI (T0) | **0.68** | **<0.001** | **0.63** | **<0.001** | **0.62** | **<0.001** | **0.63** | **<0.001** | **0.61** | **<0.001** |
| Neuroticism (T0) |  |  | -0.003 | 0.952 | -0.03 | 0.591 | 0.01 | 0.880 | -0.06 | 0.291 |
| Extraversion (T0) |  |  | **-0.14** | **<0.001** | **-0.13** | **0.001** | **-0.13** | **0.001** | **-0.10** | **0.015** |
| Openness (T0) |  |  | 0.04 | 0.306 | 0.04 | 0.279 | 0.04 | 0.311 | 0.04 | 0.242 |
| Agreeableness (T0) |  |  | 0.01 | 0.715 | 0.01 | 0.774 | 0.02 | 0.548 | 0.02 | 0.637 |
| Conscientiousness (T0) |  |  | -0.05 | 0.156 | -0.03 | 0.457 | -0.06 | 0.094 | -0.01 | 0.806 |
| RS-25 (T0) |  |  |  |  | -0.06 | 0.294 |  |  | **-0.16** | **0.012** |
| Change in RS-25 (T0-T1) |  |  |  |  |  |  | **-0.12** | **0.001** | **-0.15** | **<0.001** |
| *R^2^* / *R^2^* adjusted | 0.460 / 0.458 | | 0.484 / 0.477 | | 0.485 / 0.477 | | 0.497 / 0.489 | | 0.505 / 0.496 | |
|  | CD-RISC (*N* = 448) | | | | | | | | | |
|  | Step 1 | | Step 2 | | Step 3a | | Step 3b | | Step 4 | |
| Predictors | β | *p* | β | *p* | β | *p* | β | *p* | β | *p* |
| GSI (T0) | **0.68** | **<0.001** | **0.63** | **<0.001** | **0.63** | **<0.001** | **0.63** | **<0.001** | **0.62** | **<0.001** |
| Neuroticism (T0) |  |  | -0.003 | 0.952 | -0.003 | 0.949 | 0.02 | 0.703 | -0.01 | 0.793 |
| Extraversion (T0) |  |  | **-0.14** | **<0.001** | **-0.14** | **0.001** | **-0.14** | **<0.001** | **-0.11** | **0.011** |
| Openness (T0) |  |  | 0.04 | 0.306 | 0.04 | 0.309 | 0.04 | 0.302 | 0.04 | 0.232 |
| Agreeableness (T0) |  |  | 0.01 | 0.715 | 0.01 | 0.719 | 0.01 | 0.785 | 0.003 | 0.943 |
| Conscientiousness (T0) |  |  | -0.05 | 0.156 | 0.05 | 0.184 | -0.05 | 0.212 | -0.03 | 0.513 |
| CD-RISC (T0) |  |  |  |  | -0.001 | 0.981 |  |  | -0.08 | 0.173 |
| Change in CD-RISC (T0-T1) |  |  |  |  |  |  | **-0.11** | **0.001** | **-0.13** | **<0.001** |
| *R^2^* / *R^2^* adjusted | 0.460 / 0.458 | | 0.484 / 0.477 | | 0.484 / 0.476 | | 0.496 / 0.488 | | 0.498 / 0.489 | |
|  | BRS (*N* = 448) | | | | | | | | | |
|  | Step 1 | | Step 2 | | Step 3a | | Step 3b | | Step 4 | |
| Predictors | β | *p* | β | *p* | β | *p* | β | *p* | β | *p* |
| GSI (T0) | **0.68** | **<0.001** | **0.63** | **<0.001** | **0.62** | **<0.001** | **0.63** | **<0.001** | **0.61** | **<0.001** |
| Neuroticism (T0) |  |  | -0.003 | 0.952 | -0.03 | 0.583 | 0.01 | 0.921 | -0.06 | 0.225 |
| Extraversion (T0) |  |  | **-0.14** | **<0.001** | **-0.14** | **0.001** | **-0.15** | **<0.001** | **-0.13** | **0.001** |
| Openness (T0) |  |  | 0.04 | 0.306 | 0.04 | 0.299 | 0.04 | 0.289 | 0.04 | 0.259 |
| Agreeableness (T0) |  |  | 0.01 | 0.715 | 0.01 | 0.817 | 0.02 | 0.671 | 0.003 | 0.935 |
| Conscientiousness (T0) |  |  | -0.05 | 0.156 | -0.05 | 0.188 | -0.05 | 0.194 | -0.04 | 0.333 |
| BRS (T0) |  |  |  |  | -0.05 | 0.284 |  |  | **-0.15** | **0.008** |
| Change in BRS (T0-T1) |  |  |  |  |  |  | **-0.09** | **0.006** | **-0.14** | **<0.001** |
| *R^2^* / *R^2^* adjusted | 0.460 / 0.458 | | 0.484 / 0.477 | | 0.485 / 0.477 | | 0.493 / 0.484 | | 0.501 / 0.492 | |

Supplementary Table 6 Cont.

|  | BRCS (*N* = 447) | | | | | | | | | |
| --- | --- | --- | --- | --- | --- | --- | --- | --- | --- | --- |
|  | Step 1 | | Step 2 | | Step 3a | | Step 3b | | Step 4 | |
| Predictors | β | *p* | β | *p* | β | *p* | β | *p* | β | *p* |
| GSI (T0) | **0.68** | **<0.001** | **0.63** | **<0.001** | **0.63** | **<0.001** | **0.63** | **<0.001** | **0.63** | **<0.001** |
| Neuroticism (T0) |  |  | -0.003 | 0.952 | 0.02 | 0.721 | -0.002 | 0.967 | 0.01 | 0.807 |
| Extraversion (T0) |  |  | **-0.14** | **<0.001** | **-0.16** | **0.000** | **-0.14** | **0.000** | **-0.15** | **<0.001** |
| Openness (T0) |  |  | 0.04 | 0.306 | 0.02 | 0.528 | 0.03 | 0.346 | 0.03 | 0.482 |
| Agreeableness (T0) |  |  | 0.01 | 0.715 | 0.01 | 0.794 | 0.01 | 0.849 | 0.01 | 0.846 |
| Conscientiousness (T0) |  |  | -0.05 | 0.156 | -0.07 | 0.073 | -0.05 | 0.166 | -0.07 | 0.105 |
| BRCS (T0) |  |  |  |  | 0.07 | 0.078 |  |  | 0.05 | 0.319 |
| Change in BRCS (T0-T1) |  |  |  |  |  |  | -0.06 | 0.096 | -0.03 | 0.429 |
| *R^2^* / *R^2^* adjusted | 0.460 / 0.458 | | 0.484 / 0.477 | | 0.487 / 0.479 | | 0.487 / 0.479 | | 0.488 / 0.479 | |

BRCS, Brief Resilience Coping Scale; BRS, Brief Resilience Scale; CD-RISC, Connor-Davidson Resilience Scale; GSI, Global Severity Index of the Brief Symptom Inventory (BSI-18) as an indicator of psychological distress; RS-25, Resilience Scale.

# Supplementary Table 7. Model indices of hierarchical multiple regression analyses for **residual change scores in psychological distress (Global Severity Index of BSI-18)** with personality traits at T0 (model 1), resilience at T0 (model 2a) or/and changes in resilience from T0 to T1 (models 2b and 3) as explanatory covariates

|  | RS-25 (*N* = 448) | | | | | | | |
| --- | --- | --- | --- | --- | --- | --- | --- | --- |
|  | Step 1 | | Step 2a | | Step 2b | | Step 3 | |
| Predictors | β | *p* | β | *p* | β | *p* | β | *p* |
| Neuroticism (T0) | -0.06 | 0.316 | -0.08 | 0.216 | -0.04 | 0.450 | -0.13 | 0.064 |
| Extraversion (T0) | **-0.19** | **<0.001** | **-0.18** | **0.001** | **-0.18** | **0.001** | **-0.14** | **0.011** |
| Openness (T0) | 0.05 | 0.338 | 0.05 | 0.321 | 0.04 | 0.343 | 0.05 | 0.287 |
| Agreeableness (T0) | 0.03 | 0.586 | 0.02 | 0.614 | 0.04 | 0.435 | 0.03 | 0.475 |
| Conscientiousness (T0) | -0.07 | 0.166 | -0.05 | 0.381 | -0.08 | 0.100 | -0.02 | 0.680 |
| RS-25 (T0) |  |  | -0.06 | 0.468 |  |  | **-0.18** | **0.032** |
| Change in RS-25 (T0-T1) |  |  |  |  | **-0.16** | **0.001** | **-0.20** | **<0.001** |
| *R^2^* / *R^2^* adjusted | 0.039 / 0.028 | | 0.040 / 0.027 | | 0.065 / 0.052 | | 0.074 /0.060 | |
|  | CD-RISC (*N* = 448) | | | | | | | |
|  | Step 1 | | Step 2a | | Step 2b | | Step 3 | |
| Predictors | β | *p* | β | *p* | β | *p* | β | *p* |
| Neuroticism (T0) | -0.06 | 0.316 | -0.05 | 0.460 | -0.03 | 0.617 | -0.07 | 0.309 |
| Extraversion (T0) | **-0.19** | **<0.001** | **-0.20** | **0.001** | **-0.19** | **0.000** | **-0.16** | **0.008** |
| Openness (T0) | 0.05 | 0.338 | 0.04 | 0.352 | 0.05 | 0.334 | 0.05 | 0.275 |
| Agreeableness (T0) | 0.03 | 0.586 | 0.03 | 0.574 | 0.02 | 0.650 | 0.02 | 0.763 |
| Conscientiousness (T0) | -0.07 | 0.166 | -0.08 | 0.171 | -0.06 | 0.224 | -0.04 | 0.474 |
| CD-RISC (T0) |  |  | 0.01 | 0.851 |  |  | -0.09 | 0.264 |
| Change in CD-RISC (T0-T1) |  |  |  |  | **-0.15** | **0.001** | **-0.17** | **0.001** |
| *R^2^* / *R^2^* adjusted | 0.039 / 0.028 | | 0.039 / 0.026 | | 0.062 / 0.049 | | 0.064 / 0.049 | |
|  | BRS (*N* = 448) | | | | | | | |
|  | Step 1 | | Step 2a | | Step 2b | | Step 3 | |
| Predictors | β | *p* | β | *p* | β | *p* | β | *p* |
| Neuroticism (T0) | -0.06 | 0.316 | -0.08 | 0.217 | -0.05 | 0.422 | -0.14 | 0.048 |
| Extraversion (T0) | **-0.19** | **<0.001** | **-0.19** | **0.001** | **-0.20** | **0.000** | **-0.18** | **0.001** |
| Openness (T0) | 0.05 | 0.338 | 0.05 | 0.336 | 0.05 | 0.319 | 0.05 | 0.302 |
| Agreeableness (T0) | 0.03 | 0.586 | 0.02 | 0.639 | 0.03 | 0.548 | 0.02 | 0.714 |
| Conscientiousness (T0) | -0.07 | 0.166 | -0.07 | 0.189 | -0.07 | 0.205 | -0.05 | 0.326 |
| BRS (T0) |  |  | -0.05 | 0.472 |  |  | **-0.16** | **0.025** |
| Change in BRS (T0-T1) |  |  |  |  | **-0.13** | **0.006** | **-0.18** | **0.001** |
| *R^2^* / *R^2^* adjusted | 0.039 / 0.028 | | 0.040 / 0.027 | | 0.056 / 0.043 | | 0.066 / 0.052 | |
|  | BRCS (*N* = 447) | | | | | | | |
|  | Step 1 | | Step 2a | | Step 2b | | Step 3 | |
| Predictors | β | *p* | β | *p* | β | *p* | β | *p* |
| Neuroticism (T0) | -0.06 | 0.316 | -0.03 | 0.629 | -0.06 | 0.322 | -0.04 | 0.538 |
| Extraversion (T0) | **-0.19** | **<0.001** | **-0.21** | **0.000** | **-0.19** | **0.000** | **-0.20** | **0.000** |
| Openness (T0) | 0.05 | 0.338 | 0.03 | 0.567 | 0.04 | 0.381 | 0.03 | 0.524 |
| Agreeableness (T0) | 0.03 | 0.586 | 0.02 | 0.658 | 0.02 | 0.711 | 0.02 | 0.709 |
| Conscientiousness (T0) | -0.07 | 0.166 | -0.09 | 0.077 | -0.07 | 0.176 | -0.09 | 0.112 |
| BRCS (T0) |  |  | 0.10 | 0.078 |  |  | 0.07 | 0.318 |
| Change in BRCS (T0-T1) |  |  |  |  | -0.08 | 0.099 | -0.05 | 0.436 |
| *R^2^* / *R^2^* adjusted | 0.039 / 0.028 | | 0.046 / 0.033 | | 0.045 / 0.032 | | 0.047 / 0.032 | |

BRCS, Brief Resilience Coping Scale; BRS, Brief Resilience Scale; CD-RISC, Connor-Davidson Resilience Scale; RS-25, Resilience Scale.

# Supplementary Table 8. Model indices of hierarchical multiple regression analyses for **psychological distress (Global Severity Index of BSI-18) at T1** with psychological distress at T0 (model 1), resilience at T0 (model 2a) or/and changes in resilience from T0 to T1 (models 2b and 3) as explanatory covariates

|  | RS-25 (*N* = 448) | | | | | | | |
| --- | --- | --- | --- | --- | --- | --- | --- | --- |
|  | Step 1 | | Step 2a | | Step 2b | | Step 3 | |
| Predictors | β | *p* | β | *p* | β | *p* | β | *p* |
| GSI (T0) | **0.68** | **<0.001** | **0.61** | **<0.001** | **0.69** | **<0.001** | **0.59** | **<0.001** |
| RS-25 (T0) |  |  | **-0.14** | **<0.001** |  |  | **-0.20** | **<0.001** |
| Change in RS-25 (T0-T1) |  |  |  |  | **-0.14** | **<0.001** | **-0.18** | **<0.001** |
| *R^2^* / *R^2^* adjusted | 0.468 / 0.467 | | 0.483 / 0.481 | | 0.487 / 0.485 | | 0.514 / 0.511 | |
|  | CD-RISC (*N* = 448) | | | | | | | |
|  | Step 1 | | Step 2a | | Step 2b | | Step 3 | |
| Predictors | β | *p* | β | *p* | β | *p* | β | *p* |
| GSI (T0) | **0.68** | **<0.001** | **0.63** | **<0.001** | **0.69** | **<0.001** | **0.62** | **<0.001** |
| CD-RISC (T0) |  |  | **-0.12** | **0.003** |  |  | **-0.17** | **<0.001** |
| Change in CD-RISC (T0-T1) |  |  |  |  | **-0.11** | **0.001** | **-0.16** | **<0.001** |
| *R^2^* / *R^2^* adjusted | 0.468 / 0.467 | | 0.479 / 0.477 | | 0.481 / 0.479 | | 0.501 / 0.498 | |
|  | BRS (*N* = 448) | | | | | | | |
|  | Step 1 | | Step 2a | | Step 2b | | Step 3 | |
| Predictors | β | *p* | β | *p* | β | *p* | β | *p* |
| GSI (T0) | **0.68** | **<0.001** | **0.64** | **<0.001** | **0.69** | **<0.001** | **0.61** | **<0.001** |
| BRS (T0) |  |  | **-0.10** | **0.011** |  |  | **-0.17** | **<0.001** |
| Change in BRS (T0-T1) |  |  |  |  | **-0.08** | **0.017** | **-0.14** | **<0.001** |
| *R^2^* / *R^2^* adjusted | 0.468 / 0.467 | | 0.476 / 0.474 | | 0.475 / 0.473 | | 0.493 / 0.490 | |
|  | BRCS (*N* = 447) | | | | | | | |
|  | Step 1 | | Step 2a | | Step 2b | | Step 3 | |
| Predictors | β | *p* | β | *p* | β | *p* | β | *p* |
| GSI (T0) | **0.68** | **<0.001** | **0.68** | **<0.001** | **0.68** | **<0.001** | **0.66** | **<0.001** |
| BRCS (T0) |  |  | -0.03 | 0.482 |  |  | -0.08 | 0.058 |
| Change in BRCS (T0-T1) |  |  |  |  | **-0.07** | **0.043** | **-0.11** | **0.007** |
| *R^2^* / *R^2^* adjusted | 0.468 / 0.467 | | 0.469 / 0.466 | | 0.473 / 0.471 | | 0.477 / 0.474 | |

BRCS, Brief Resilience Coping Scale; BRS, Brief Resilience Scale; CD-RISC, Connor-Davidson Resilience Scale; GSI, Global Severity Index of the Brief Symptom Inventory (BSI-18) as an indicator of psychological distress; RS-25, Resilience Scale.

# Supplementary Table 9. Model indices of hierarchical multiple regression analyses for **psychological distress (Global Severity Index of BSI-18) at T1** with personality traits at T0 (model 1), resilience at T0 (model 2a) or/and changes in resilience from T0 to T1 (models 2b and 3) as explanatory covariates

|  | RS-25 (*N* = 448) | | | | | | | |
| --- | --- | --- | --- | --- | --- | --- | --- | --- |
|  | Step 1 | | Step 2a | | Step 2b | | Step 3 | |
| Predictors | β | *p* | β | *p* | β | *p* | β | *p* |
| Neuroticism (T0) | **0.34** | **<0.001** | **0.23** | **<0.001** | **0.35** | **<0.001** | **0.20** | **0.001** |
| Extraversion (T0) | **-0.15** | **0.003** | **-0.10** | **0.038** | **-0.14** | **0.004** | -0.07 | 0.181 |
| Openness (T0) | 0.06 | 0.198 | 0.06 | 0.146 | 0.05 | 0.200 | 0.06 | 0.124 |
| Agreeableness (T0) | -0.04 | 0.328 | -0.05 | 0.250 | -0.04 | 0.422 | -0.04 | 0.336 |
| Conscientiousness (T0) | -0.06 | 0.176 | 0.01 | 0.810 | -0.07 | 0.121 | 0.04 | 0.465 |
| RS-25 (T0) |  |  | **-0.21** | **0.002** |  |  | **-0.33** | **<0.001** |
| Change in RS-25 (T0-T1) |  |  |  |  | **-0.11** | **0.008** | **-0.18** | **<0.001** |
| *R^2^* / *R^2^* adjusted | 0.216 / 0.207 | | 0.232 / 0.221 | | 0.228 / 218 | | 0.261 / 0.249 | |
|  | CD-RISC (*N* = 448) | | | | | | | |
|  | Step 1 | | Step 2a | | Step 2b | | Step 3 | |
| Predictors | β | p | β | p | β | p | β | p |
| Neuroticism (T0) | **0.34** | **<0.001** | **0.29** | **<0.001** | **0.36** | **<0.001** | **0.27** | **<0.001** |
| Extraversion (T0) | **-0.15** | **0.003** | **-0.11** | **0.032** | **-0.14** | **0.004** | -0.08 | 0.157 |
| Openness (T0) | 0.06 | 0.198 | 0.06 | 0.146 | 0.06 | 0.195 | 0.07 | 0.104 |
| Agreeableness (T0) | -0.04 | 0.328 | -0.05 | 0.252 | -0.05 | 0.288 | -0.06 | 0.154 |
| Conscientiousness (T0) | -0.06 | 0.176 | -0.04 | 0.438 | -0.06 | 0.225 | -0.01 | 0.908 |
| CD-RISC (T0) |  |  | -0.11 | 0.124 |  |  | **-0.20** | **0.006** |
| Change in CD-RISC (T0-T1) |  |  |  |  | **-0.11** | **0.009** | **-0.16** | **0.001** |
| *R^2^* / *R^2^* adjusted | 0.216 / 0.207 | | 0.220 / 0.209 | | 0.228 / 0.217 | | 0.241 / 0.229 | |
|  | BRS (*N* = 448) | | | | | | | |
|  | Step 1 | | Step 2a | | Step 2b | | Step 3 | |
| Predictors | β | p | β | p | β | p | β | p |
| Neuroticism (T0) | **0.34** | **<0.001** | **0.23** | **<0.001** | **0.34** | **<0.001** | **0.17** | **0.005** |
| Extraversion (T0) | **-0.15** | **0.003** | **-0.12** | **0.018** | **-0.15** | **0.002** | **-0.11** | **0.028** |
| Openness (T0) | 0.06 | 0.198 | 0.06 | 0.185 | 0.06 | 0.188 | 0.06 | 0.157 |
| Agreeableness (T0) | -0.04 | 0.328 | -0.06 | 0.190 | -0.04 | 0.346 | -0.06 | 0.144 |
| Conscientiousness (T0) | -0.06 | 0.176 | -0.05 | 0.284 | -0.06 | 0.206 | -0.03 | 0.491 |
| BRS (T0) |  |  | **-0.19** | **0.001** |  |  | **-0.31** | **<0.001** |
| Change in BRS (T0-T1) |  |  |  |  | **-0.09** | **0.043** | **-0.19** | **<0.001** |
| *R^2^* / *R^2^* adjusted | 0.216 / 0.207 | | 0.234 / 0.224 | | 0.223 / 0.212 | | 0.262 / 0.250 | |
|  | BRCS (*N* = 447) | | | | | | | |
|  | Step 1 | | Step 2a | | Step 2b | | Step 3 | |
| Predictors | β | p | β | p | β | p | β | p |
| Neuroticism (T0) | **0.34** | **<0.001** | **0.36** | **<0.001** | **0.34** | **<0.001** | **0.35** | **<0.001** |
| Extraversion (T0) | **-0.15** | **0.003** | **-0.16** | **0.001** | **-0.14** | **0.003** | **-0.15** | **0.002** |
| Openness (T0) | 0.06 | 0.198 | 0.04 | 0.330 | 0.05 | 0.218 | 0.05 | 0.303 |
| Agreeableness (T0) | -0.04 | 0.328 | -0.05 | 0.291 | -0.05 | 0.265 | -0.05 | 0.266 |
| Conscientiousness (T0) | -0.06 | 0.176 | -0.08 | 0.093 | -0.06 | 0.180 | -0.07 | 0.130 |
| BRCS (T0) |  |  | 0.08 | 0.145 |  |  | 0.05 | 0.432 |
| Change in BRCS (T0-T1) |  |  |  |  | -0.06 | 0.156 | -0.04 | 0.477 |
| *R^2^* / *R^2^* adjusted | 0.216 / 0.207 | | 0.219 / 0.209 | | 0.219 / 0.209 | | 0.220 / 0.208 | |

BRCS, Brief Resilience Coping Scale; BRS, Brief Resilience Scale; CD-RISC, Connor-Davidson Resilience Scale; GSI, Global Severity Index of the Brief Symptom Inventory (BSI-18) as an indicator of psychological distress; RS-25, Resilience Scale.

|  | T0 | T0 | T0 | T0 | T0 | T0 | T0 | T0 | T0 | T0 | T1 | T1 | T1 | T1 | T1 |  |
| --- | --- | --- | --- | --- | --- | --- | --- | --- | --- | --- | --- | --- | --- | --- | --- | --- |
|  | RS25 | CD-RISC | BRS | BRCS | NEOFFI  extra-version | NEOFFI neuro-ticism | NEOFFI agreeable-ness | NEOFFI conscien-tiousness | NEOFFI open-  ness | BSI | RS25 | CD-RISC | BRS | BRCS | BSI |  |
| 447 |  |  |  |  |  |  |  |  |  |  |  |  |  |  |  | 0 |
| 1 |  |  |  |  |  |  |  |  |  |  |  |  |  |  |  | 1 |
| 1 |  |  |  |  |  |  |  |  |  |  |  |  |  |  |  | 1 |
| 1 |  |  |  |  |  |  |  |  |  |  |  |  |  |  |  | 2 |
| 1 |  |  |  |  |  |  |  |  |  |  |  |  |  |  |  | 1 |
| 1 |  |  |  |  |  |  |  |  |  |  |  |  |  |  |  | 2 |
| 2 |  |  |  |  |  |  |  |  |  |  |  |  |  |  |  | 3 |
| 7 |  |  |  |  |  |  |  |  |  |  |  |  |  |  |  | 4 |
| 1 |  |  |  |  |  |  |  |  |  |  |  |  |  |  |  | 3 |
| 1 |  |  |  |  |  |  |  |  |  |  |  |  |  |  |  | 4 |
| 1 |  |  |  |  |  |  |  |  |  |  |  |  |  |  |  | 3 |
| 3 |  |  |  |  |  |  |  |  |  |  |  |  |  |  |  | 2 |
| 2 |  |  |  |  |  |  |  |  |  |  |  |  |  |  |  | 3 |
| 3 |  |  |  |  |  |  |  |  |  |  |  |  |  |  |  | 4 |
| 2 |  |  |  |  |  |  |  |  |  |  |  |  |  |  |  | 4 |
| 2 |  |  |  |  |  |  |  |  |  |  |  |  |  |  |  | 5 |
| 1 |  |  |  |  |  |  |  |  |  |  |  |  |  |  |  | 6 |
| 1 |  |  |  |  |  |  |  |  |  |  |  |  |  |  |  | 7 |
| 4 |  |  |  |  |  |  |  |  |  |  |  |  |  |  |  | 8 |
| 1 |  |  |  |  |  |  |  |  |  |  |  |  |  |  |  | 7 |
| 1 |  |  |  |  |  |  |  |  |  |  |  |  |  |  |  | 8 |
| 2 |  |  |  |  |  |  |  |  |  |  |  |  |  |  |  | 7 |
| 1 |  |  |  |  |  |  |  |  |  |  |  |  |  |  |  | 8 |
| 1 |  |  |  |  |  |  |  |  |  |  |  |  |  |  |  | 8 |
|  | 1 | 5 | 6 | 6 | 14 | 14 | 14 | 15 | 14 | 12 | 15 | 12 | 16 | 14 | 25 |  |

Supplementary Figure 1. Missing data pattern in the study population included at T0 (*N* = 488). T0 denotes the first measurement time point and t1 the second measurement time-point. Numbers on the left side indicate the frequency with which this missing data pattern occurs. Numbers in the bottom indicate the number of missing data of the respective variable. Numbers on the right side indicate the number of missing variables in the respective missing data pattern. Color coding: green, not missing; red, missing. Abbreviations: BRCS, Brief Resilience Coping Scale; BRS, Brief Resilience Scale; BSI, Brief Symptom Inventory; CD-RISC, Connor-Davidson Resilience Scale; NEOFFI, NEO Five Factors Inventory; RS-25, Resilience Scale.
